# Polyphenol-rich extract of *Pimenta dioica* berries (Allspice) kills breast cancer cells by autophagy and delays growth of triple negative breast cancer in athymic mice

**DOI:** 10.18632/oncotarget.3834

**Published:** 2015-04-14

**Authors:** Lei Zhang, Nagarajarao Shamaladevi, Guddadarangavvanahally K. Jayaprakasha, Bhimu S. Patil, Bal L. Lokeshwar

**Affiliations:** ^1^ Sheila and David Fuente Graduate Program in Cancer Biology, Sylvester Comprehensive Cancer Center, Miller School of Medicine, University of Miami, Miami, Florida, USA; ^2^ Departments of Urology and Radiation Oncology, Miller School of Medicine, University of Miami, Miami, Florida, USA; ^3^ Vegetable and Fruit Improvement Center, Department of Horticultural Sciences, Texas A&M University, College Station, Texas, USA; ^4^ Research Service, Bruce Carter Memorial Veterans Health Administration Medical Center, Miami, Florida, USA

**Keywords:** breast cancer, chemoprevention, chemo dietary-cancer prevention, autophagy, mTOR signaling

## Abstract

Bioactive compounds from edible plants have limited efficacy in treating advanced cancers, but they have potential to increase the efficacy of chemotherapy drugs in a combined treatment. An aqueous extract of berries of Pimenta dioica (Allspice) shows promise as one such candidate for combination therapy or chemoprevention. An aqueous extract of Allspice (AAE) was tested against human breast cancer (BrCa) cells *in vitro* and *in vivo*. AAE reduced the viability and clonogenic growth of several types of BrCa cells (IC_50_ ≤ 100 μg/ml) with limited toxicity in non-tumorigenic, quiescent cells (IC_50_ >200 μg/ml). AAE induced cytotoxicity in BrCa was inconsistent with apoptosis, but was associated with increased levels of autophagy markers LC3B and LC3B-positive puncta. Silencing the expression of autophagy related genes (ATGs) prevented AAE-induced cell death. Further, AAE caused inhibition of Akt/mTOR signaling, and showed enhanced cytotoxicity when combined with rapamycin, a chemotherapy drug and an inhibitor of mTOR signaling. Oral administration (gavage) of AAE into athymic mice implanted with MDA-MB231 tumors inhibited tumor growth slightly but not significantly (mean decrease ~ 14%, *p* ≥ 0.20) if mice were gavaged post-tumor implant. Tumor growth showed a significant delay (38%) in tumor palpability and growth rate (time to reach tumor volume ≥ 1,000 mm^3^) when mice were pre-dosed with AAE for two weeks. Analysis of tumor tissues showed increased levels of LC3B in AAE treated tumors, indicating elevated autophagic tumor cell death *in vivo* in treated mice. These results demonstrate antitumor and chemo-preventive activity of AAE against BrCa and potential for adjuvant to mTOR inhibition.

## INTRODUCTION

Breast cancer (BrCa), the most common cancer among women cancer survivors, is expected to be the first in incidence in the United States within a decade [1]. Despite significant improvements in treating the cancer, many women who received adjuvant chemotherapy for their early-stage breast cancer do not benefit significantly [2]. For women with metastatic breast cancer, chemotherapy is usually necessary but is often a palliative treatment [3].

Most cytotoxic, chemotherapeutic drugs used against solid tumors induce tumor cell death by one or more of the three common mechanisms: cell cycle arrest by inhibiting DNA synthesis, necrotic cell death, and/or direct apoptosis [4, 5]. Since these drugs also cause irreversible cardiac, hepatic, hematopoietic and neuromuscular damages, their use is often limited as the salvage therapy or as the last therapeutic option that are increasingly regarded as palliative rather than curative intent. Recently, more targeted therapeutics that block cell survival pathways such as mTOR mediated pathway have emerged [6]. Although, this class of drugs such as rapamycin and its derivatives called rapalogs have been well tolerated, their efficacy is limited [7]. Increasing the efficacy of this “new” classes of drugs with natural therapeutics, which generally show limited normal tissue toxicity, could significantly enhance BrCa therapy. Since many of the natural products act as chemo preventive agents by diminishing the aggressiveness of tumor cells, they may provide ideal adjuvants, or neo adjuvants to more aggressive tumors.

Inactivation of mTOR activity can suppress cell growth to reduce energy demand and induces autophagy for stress adaptation [8]. Autophagy is an evolutionarily conserved process that cell engulfs cytoplasmic material within a vacuole and delivers it to the lysosome for degradation. This process not only eliminates damaged organelles or misfolded proteins as a quality control mechanism, but also generates amino acids and small molecules that are shuttled back for reuse as building blocks for proteins and nucleic acids. It contributes to basal cellular and tissue homeostasis, developmental regulations [9] and further helps cells to cope with metabolic stress such as nutrition deprivation, hypoxia or pathogen infection [10]. Autophagy appears to serve as a prosurvival mechanism under many conditions [11, 12]. However, the finding that defects in the autophagic machinery accelerate oncogenesis indicated the potential oncosuppressive role of autophagy. Recent evidence has pointed out an anti-survival, thus tumor suppressive role of autophagy [13, 14]. More recent reports suggest autophagy as a tumor-suppressing mechanism by restricting Ras-induced oncogenesis [15], negatively regulating cancer invasiveness [16] and suppressing renal tumorigenesis [17]. However, this mechanism is still unsettled in cancer therapy as increased, irreversible autophagy may indeed be a novel mechanism to kill tumor cells. A dietary compound induced autophagy of tumor cells might help enhance mTOR inhibitors to accelerate tumor cell killing and thus herald a novel cancer therapy.

Earlier we reported the identification of the Aqueous Allspice Extract (AAE) as an anti-cancer formulation against prostate cancer and identified a potent novel anti-proliferative compound Ericifolin (Eugenol 5-O-β-galloylglucopyranoside) from AAE that inhibits transcription of androgen receptor mRNA [18]. An attempt to extend this finding to breast cancer was undertaken to identify AAE or Ericifolin as a potential anti-BrCa agent. Since both prostate and breast cancers are hormone driven diseases with similar progression pattern (hormone dependence, hormone independent-progression etc.) we hypothesized that AAE/Ericifolin should inhibit BrCa cell proliferation and prevent tumor incidence/growth.

In this article, we show potent cytotoxic and anti-tumor activities of AAE in BrCa cells but the mechanisms by which tumor cells are killed and tumor growth is halted are completely different from that observed in prostate cancer models treated with AAE or Ericifolin. Evidence is presented to show that the tumor cell death is due to induction of autophagy and a lack of apoptosis or cell cycle arrest as a basis of its antitumor activity. Further, we demonstrate both chemo-preventive and antitumor activity of orally administered (gavage) AAE in the MDA-MB231 (MB231) BrCa model in athymic mice. Importantly, these effects, unlike the known drug-induced anticancer mechanisms, such as cell cycle arrest or apoptosis that we found in prostate cancer, were driven by autophagy both *in vitro* and *in vivo* BrCa models and further, we show that Ericifolin has limited activity against BrCa cells.

## RESULTS

### AAE is cytotoxic to BrCa cells but does not affect cell cycle

**Figure 1:**
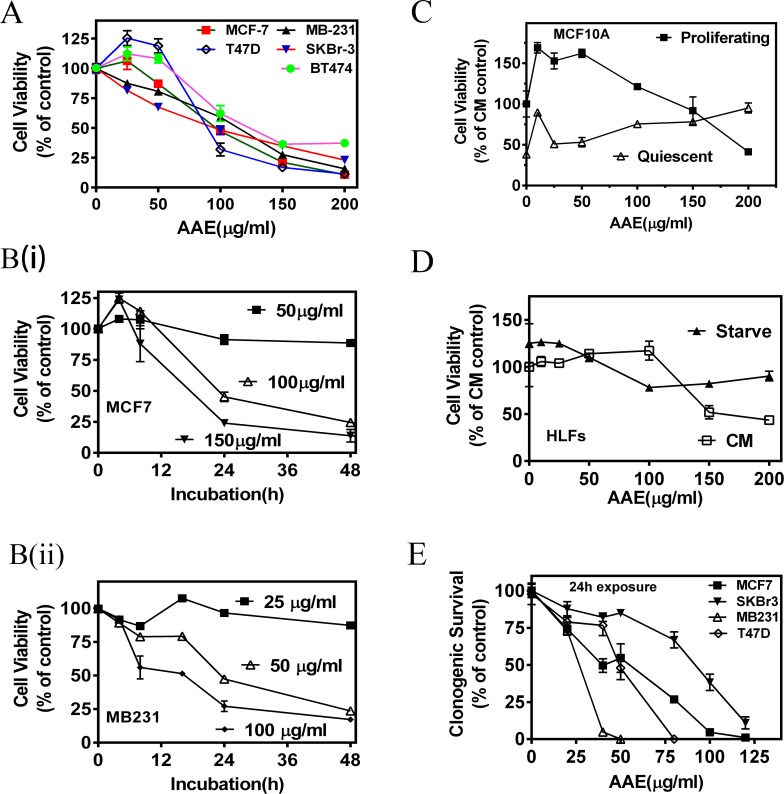
AAE inhibits BrCa cell viability and replication potential (colony-forming ability) **A.** Dose-dependent decrease in viability of BrCa cells exposed to AAE for 72h. MTT assay was used to determine percent of viable cells. Each graph was drawn from data pooled from three independent experiments containing optical densities of three triplicate culture wells treated with AAE. Points shown are Mean ± Standard Error (SE). CM=complete medium **B.** Exposure time-dependent decrease in viability of BrCa cells with AAE. Culture wells received indicated amount of AAE for indicated duration. MTT assay was used to determine viability at the end of 48h. B (i): Viability of MCF7 cells; B (ii): Viability of MB231 cells. **C.** Normal (non-tumorigenic) breast epithelial cells are resistant to cytotoxicity by AAE. Cytotoxicity of AAE on proliferating and quiescent normal breast epithelial cell cultures (MCF10A). MCF10A cells cultured for 24h with or without growth factor containing basal medium [MEBM ± Growth Factors Mix (Single-Quots, Lonza Inc., Allendale, NJ)] were exposed to AAE for 72h and surviving MCF10A cells were estimated using MTT assay [58]. **D.** Cytotoxicity of AAE on serum-starved and non-starved Human Lung Fibroblasts (HLFs). About 5 × 10^3^ HLFs/well/48well clustures were cultured in in CM or RPMI + 0.1%BSA for starvation. Cells were then treated with AAE for 72h and viability quantified by MTT assay. **E.** AAE reduces clonogenic potential of BrCa cells when treated at low density. BrCa cells plated at low density (0.5 – 1 × 10^3^ cells/well/2ml) in 6-well plates were incubated with several concentrations of AAE for 24h. Culture wells were then changed to normal culture medium without AAE and surviving cells were allowed to form colonies for 7-10 days. Data shown in Panel A-E are mean ± SEM, *n* ≥ 3.

We observed a significant decrease in population of established BrCa cells (MCF7, SKBR3, MDA-MB231, T47D and BT474) incubated with AAE over a 72h period. As shown in Figure 1A, the 50 % inhibition dose calculated from the MTT assay, varied from 50 μg/ml to 100 μg/ml among the BrCa cell lines tested. AAE induced cytotoxicity was also dependent on duration of incubation suggesting a continuous cytotoxicity on susceptible cells (Figure 1B). As compared to other BrCa cell lines, the estrogen receptor (ER), progesterone receptor (PR) and HER2 non-expressing (Triple negative) MB231 cells showed more sensitivity to AAE. Exposing two non-tumorigenic human mammary epithelial cells (MCF-10A and MCF12A) to AAE did not significantly affect their viability, indicating a lack of cytotoxicity of AAE in normal breast epithelial cells (Figure 1C, Figure S1A). Furthermore, viability was unaltered when AAE was tested on a human lung fibroblast cell line made quiescent by serum-starvation (Figure 1D).

The ability of AAE to render BrCa cells without proliferative (clonogenic or colony -forming) potential, was examined by colony-formation assay. Dose-dependent inhibition of colony formation indicated that AAE significantly diminishes clonogenic potential of BrCa cells. Colony assays also demonstrated MB231 cells are more sensitive to AAE than MCF7 cells, with 50 % inhibition of colony formation at 25μg/ml and 50 μg/ml, respectively (Figure 1E).

We tested whether AAE induced cytotoxicity is due to its alteration of cell cycle phase-progression by flow-cytometry [19]. Determining the cell cycle phase distribution of BrCa cells incubated with AAE for 24-48h revealed no significant changes in any of the cell cycle phases, G0/G1, S and G2/M, respectively (Figure S1B).

### AAE-induced cell death is not associated with characteristics of apoptotic pathway in BrCa cells

**Figure 2:**
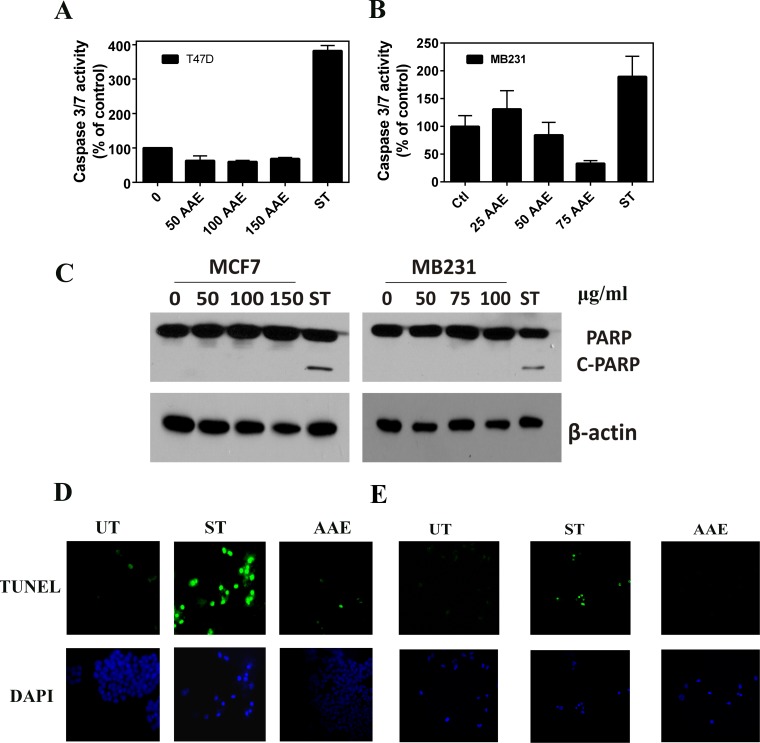
AAE does not induce apoptosis in BrCa cells **A & B.** Lack of cell death caspase (Caspase3/7) activation by AAE in T47D and MB321 cells. T47D and MB231 cell cultures in 96-well clusters were treated with 50-150 μg/ml AAE for 24h. Duplicate wells are also treated with 1μM staurosporine, a known inducer of caspase 3/7 in these cells. Caspase 3/7 activity was measured by Caspase-Glo 3/7 assay systems (Promega, Madison, WI). Data shown are Mean ± SEM, *n* = 6. **C.** Lack of cleaved-PARP in BrCa cells treated with AAE for 24h. Cells (1.0 × 10^4^ cells/well) seeded in 12-well plates were treated with indicated amount of AAE for 24h. Cleaved PARP levels were detected using western blots of cell lysates from indicated cultures. **D & E.** Apoptosis determined by TUNEL assay of 24h AAE-treated MCF7 (**D**) and MB231 (**E**) BrCa cells. Green (FITC) TUNEL-positive cells and DAPI images (630X 600x). Results from a typical experiment is shown. Similar results were obtained from two more repeats. UT: untreated, ST cells: 1 μM staurosporine.

Since apoptosis is the most common type of cell death induced by antitumor agents, we examined the characteristics of apoptosis in AAE treated BrCa cells. To detect early apoptosis, we determined the binding of EGFP-annexin-V to externalized plasma membrane phosphatidyl serine, and changes in mitochondrial membrane potential using the pH sensitive dye JC-1 fluorescence in AAE-treated or control (untreated) MCF7 cells. As shown in Figure S1C, we did not observe significant alteration in mitochondrial depolarization or their permeability in MCF7 cells exposed to AAE. Further, we assayed the activation of cell death related caspases using a caspase 3/7 activity kit. As shown in Figure 2A & 2B, we were unable to detect significant change in the levels of active caspase 3/7 in two BrCa cell lines tested, namely, T47D (ER-positive) and MB231 upon exposure to AAE up to 72h. Since caspase-mediated apoptosis in MCF7 cells is mediated by unusual caspase repertoire, we used T47D and MB231 BrCa cells to test potential caspase-mediated apoptotic pathway induced by AAE in BrCa [20]. Further, AAE did not induce the cleavage of PARP in BrCa cells compared to that induced by staurosporine [21] (Figure 2C).

DNA fragmentation is a key feature during the late stages of apoptosis, and is usually detected by Terminal deoxynucleotidyl transferase dUTP Nick-End Labeling (TUNEL) assay [22]. As shown in Figure 2D & 2E, fragmented DNA that has been end labeled with fluorescent nucleotide (appears as green fluorescent nuclei) was only observed in cultures treated with staurosporine (1μM) but not in cultures treated with AAE. These data suggested that AAE-induced cell death is not likely due to apoptosis.

### AAE-induced cell death is associated with the activation of the autophagy pathway in BrCa cells

**Figure 3:**
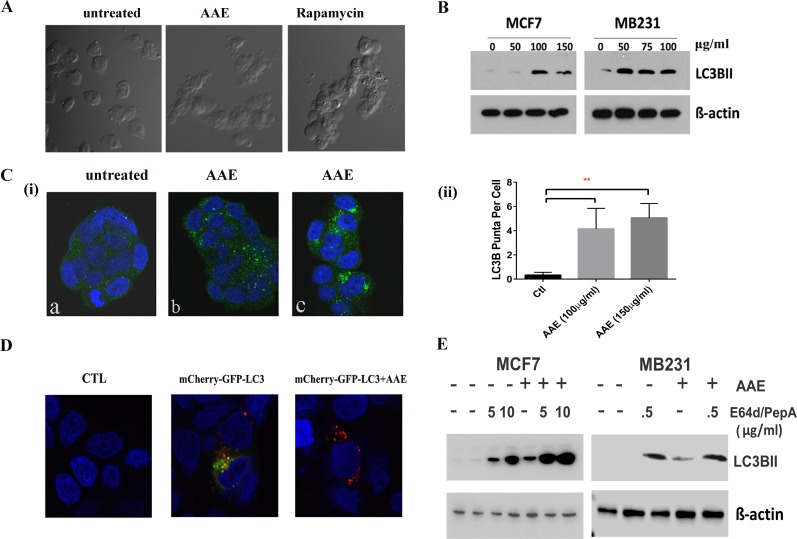
AAE induced autophagic cell death in BrCa cells **A.** Morphological features of autophagy in AAE treated MB231 cells as seen under phase-contrast microscopy. Magnification: 100X. Cells became vacuolated after AAE or rapamycin treatment for 24h. **B.** AAE induced autophagy in BrCa cells. Increased levels of LC3B protein in BrCa cells treated with AAE. C.(i). Immunofluorescence detection of LC3B puncta induction in AAE treated BrCa cells. Untreated cells are shown in (a) and AAE treated cells are shown in (b: 100 μg/ml) and (c: 150 μg/ml). Micrograph magnification: 630X. **C.** (ii): Quantification of number of puncta/cell in treated and control cultures. There was a significant (**p < 0.05) increase in LC3B puncta in treated cells as compared to that of control. **D.** Increased levels of autolysosomes in AAE treated-cells as visualized by red-fluorescence (mCherry) and quenching of GFP in acidic compartment of lysosomes resulting from fusion of autophagosome and lysosome. Confocal microscopy was used to obtain multicolor micrographs (Magnification: 630X); **E.** AAE induced autophagic flux in BrCa cells. BrCa cells were incubated with lysosomal inhibitors pepstatin A and E64d (5 μg/ml and 10 μg/ml each, for MCF-7 cells and 0.5 μg/ml for MB231 cells) and/or AAE (100 μg/ml) for 24h. Cell lysates were analyzed by immunoblotting for LC3BII (LC3B) levels, to distinguish between autophagy induction and inhibition of autophagosome turnover.

As described above, AAE-induced cell death was not linked to either apoptosis or cell cycle arrest, we investigated whether cytotoxicity was associated with autophagy. Morphological changes in AAE-treated MB231 cells observed under a phase-contrast microscope showed high level of vacuolation after incubating cells with AAE (75μg/ml, 24h) as compared to untreated cells. This vacuolation was comparable to that observed in cells incubated with 50 nM Rapamycin for 24h (Figure 3A). To corroborate this observation further, we next analyzed the levels of the microtubule-associated protein 1 light chain 3B (LC3B), a protein associated with both the size and numbers of autophagosomes, thus a reliable marker for detecting autophagy [23]. As shown in Figure 3B, AAE treatment of both MCF7 and MB231 cell cultures caused a significant increase in the expression of LC3B. Immunofluorescence assay to visualize the endogenous LC3B in AAE treated groups showed significantly increased LC3B puncta [Figure 3C (i)]. This increase in number of LC3B containing puncta was significant at two concentrations of AAE tested [Figure 3C(ii)].

A key step of autophagic process is the formation of autolysosome. We utilized a selective in situ fluorescence-quench assay [24]. MCF7 cells were transfected with the mCherry-GFP-LC3B plasmid construct which contains cDNA for LC3B in tandem with red and green flourescence proteins mCherry and GFP, respectively. When treated with an autophagy inducing agent, increased autophagic flux results in localization of LC3B in acidic autolysosomes that quench GFP. This results in increased numbers of mCherry-positive autolysosomes. We observed increased mCherry positive but GFP-negative puncta in AAE treated BrCa cells (Figure 3D). In order to differentiate between increase of autophagic flux and deficiency in LC3B turnover, we used lysosomal inhibitors Pepstain-A and E64d to inhibit the downstream lysosomal degradation of LC3B [25]. Further increase in levels of LC3B in cells treated with both, AAE and lysosome inhibitors, indicated significant increase of AAE induced-autophagic flux (Figure 3E).

### AAE induced autophagy is rescued by depletion of ATG 5 or ATG7

**Figure 4:**
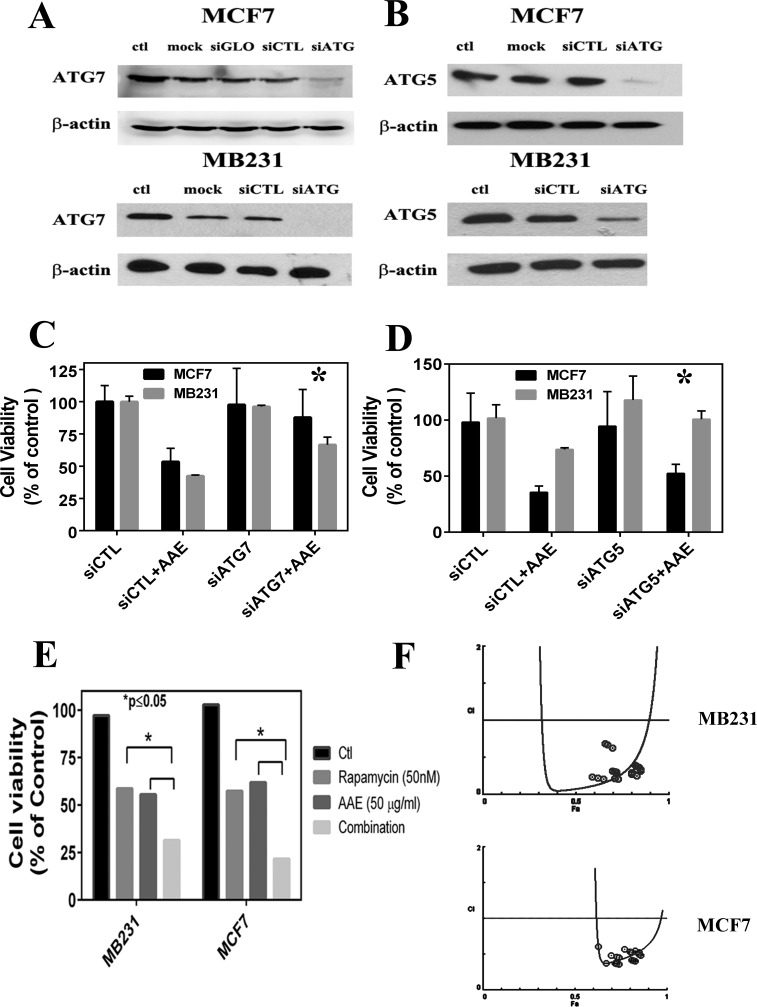
ATG knockdown blocks AAE induced autophagy and cell death **A. & B.** Immunoblotting of gene silencing by siRNA of ATG7 and ATG5 in MCF7 and MD231 cells. **C. & D.**: Percent of viable cells following various transfections with or without exposure to AAE. Transfected cells were harvested 48h after siRNA transfections, half the cell suspension used for protein estimation and immunoblotting. The other half of the harvested cells were incubated with 100 μg/ml AAE for 24h. Percent of live cells (viability) was determined after counting live and dead cells using trypan blue staining. Cell Viability was significantly rescued by autophagy inhibition of ATG7 and ATG5 compared to the background siRNA control group. **E.** Increased cytotoxicity in BrCa treated with both AAE and an autophagy inducer rapamycin. BrCa cells were treated either with AAE (25-75μg/ml) or rapamycin (10-50 nM) alone or with both drugs of various concentrations for another 72h. Cell viability was measured by MTT assay. Cell Viability decreased significantly in both treated MCF7 cells (*P < 0.05) and M231 cells (*P < 0.05) compared to AAE/rapamycin treated cells respectively. **F.** AAE and rapamycin synergistically induce cytotoxicity. The combination Index plot was generated using Compusyn (ComboSyn, Inc., Paramus, NJ, USA). Blue round circles indicate the data points in the drug combination treatment.

To confirm the key role of autophagy in AAE-induced cell death, we investigagted whether inhibition of autophagy is able to rescue cell death. Given the important role of autophagy-related-genes (ATGs) in regulating the autophagy process [26], we transiently silenced two key ATG genes, ATG7 and ATG5, in MCF7 and MB231 cells to inhibit autophagy. As shown in Figure 4A & 4B, in on-target siRNA transfected groups, the AAE induced cytotoxicity was rescued by 80 % in MCF7 cells and 50 % in MB231 cells as compared to that of siCTL transfected group. These observations indicated that inhibiting ATG7 can significantly rescue AAE induced cytotoxicity (*p* ≤ 0.05). Similar results were obtained following the ATG5 downregulation with 35 % and 38 % rescued cell death in MCF7 and MB231 cells respectively (Figure 4C & 4D). Further, since rapamycin is an established inducer of autophagy we tested the combination of AAE with rapamycin to further decrease in viability that is greater than either agents alone. We treated MCF7 and MB231 cells with several concentrations of AAE (25-75μl/ml) and rapamycin (10-50nM) separately and in combination for up to 72h, and cytotoxicity was determined by MTT assay. As shown in Figure 4E, combination treatment was significantly more cytotoxic than either agent alone (*p* < 0.05) in both BrCa cells tested. The combined efficacy, termed combination index (CI) caculated using Compusyn software showed that AAE and rapamycin work in a synergestic manner (CI < 1) as per the Chou TC and Talalay P isobologram analysis; (synergism (CI < 1), additive effect (CI = 1), and antagonism (CI > 1) [27]. The analysis performed with the tested-concentration with Fa (effect factor, cytotoxicity percentage) between (0.5,0.9) means 50 %-90 % cell viability inhibition (Figure 4F). These results suggest autophagy plays a central role in AAE induced cell death in BrCa cell lines.

### AAE down-regulates the activity of AKT-mTOR signaling pathway

**Figure 5:**
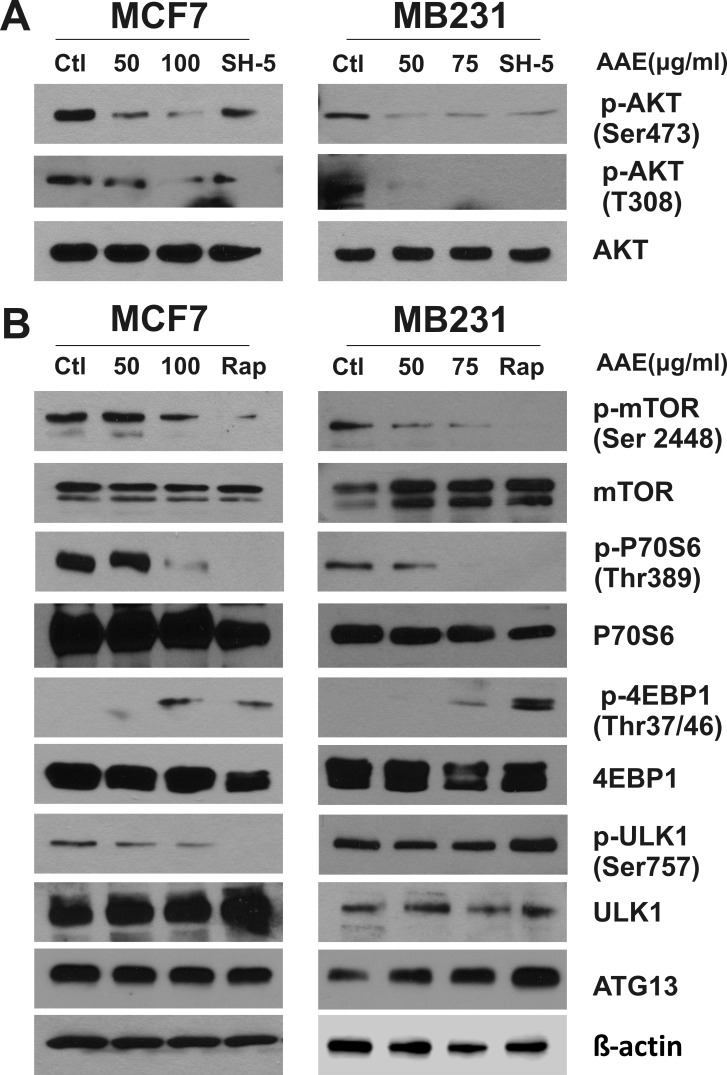
AAE down-regulates the activity of AKT-mTOR signaling pathway leading to autophagy BrCa cell cultures were treated with AAE for 24 h, cells were harvested and cell extracts were analyzed by immunoblotting. The levels of total Akt and mTOR, phospho-Akt, phospho-mTOR, total S6 ribosomal protein and 4E-BP1, phospho-S6 and phospho-4E-BP1, total ULK1 and phospho-ULK1, as well as ATG13 were detected by immunoblotting. Positive control used for panel **A** is AKT inhibitor, SH-5 at 20 μM; for panel **B** is mTOR inhibitor rapamycin at 25 nM. Levels of β-actin was used as a loading control. Since protein lysates from same experiment were analyzed for detecting signaling proteins, a single β-actin blot is shown at the bottom. All blots were repeated using new samples twice, with comparable results.

As shown in Figure 4F, AAE synergistically works with rapamycin resulting in enhanced cytotoxicity in BrCa cells potentially by increased autophagy. Mammalian target of rapamycin (mTOR) is a key regulator of signals involved with protein synthesis, cell growth and metabolism. Rapamycin is a potent mTOR Complex1 (mTORC1) inhibitor [28] and studies have shown that inhibition of mTORC1 is sufficient for autophagy induction [8]. Moreover, mTORC1 negatively regulates autophagy in a transcription-independent manner, downstream of AKT, where phosphorylated AKT at Thr308 (pAkt^−T308^) is needed [29, 30]. Therefore, we examined whether AAE induces autophagy by inhibition of AKT/mTOR signaling pathway. As shown in Figure 5A, incubation with AAE caused significant decrease of pAkt^−T308^ in both MCF7 and MB231 cells. This inhibition was comparable with that caused by treating cells with SH-5, a synthetic AKT inhibitor [31]. In addition, AAE almost abolished the phosphorylation of p-AKT^Ser473^, which is another important sites for the activity of AKT [32]. Accordingly, mTOR phosphorylation (p-mTOR ^Ser2448^) was greatly attenuated by AAE treatment, comparable to that observed upon treatment with rapamycin (Figure 5B). In parallel, AAE inhibited the activity of p70 S6 ribosomal protein and 4EBP1, which are the downstream effectors of mTOR [33]. These results are shown as decrease in p70S6 phosphorylation (Thr389) and increase in p-4EBP1 at Thr37/46 (Figure 5B). Among several proteins downstream of mTORC, the serine/threonine kinase, Atg1/ULK plays a key role in the formation of the autophagosome [34]. Recent studies have shown that mTOR regulates autophagy through direct phosphorylation of ULK1 and a complex composed of ULK1, ATG13 and FIP2000 is essential for autophagy [35, 36]. We measured the ULK1 and ATG13 expression after AAE treatment. We found marked decrease in the inactive p-ULK1 (Ser757) in MCF7 cells but no change in MB231 cells, and an increase in ATG13 in MB231 cells but no change in MCF7 cells after AAE treatment. These results suggest that inactivation of AKT/mTOR pathway by AAE treatment may be responsible for suppression of survival pathway driven by AKT.

### The biological activity of AAE in BrCa cells is not due to Ericifolin

**Figure 6:**
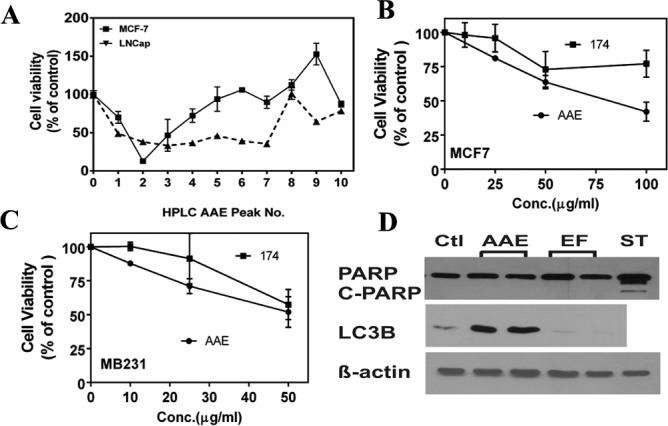
Ericifolin is not the active component in AAE against breast cancer **A.** Cytotoxicity profile of different AAE fractions tested against LNCaP prostate cancer cells and MCF7 cells. A 48-well plate was seeded with 5 × 10^3^ cells/well and treated with AAE fractions (100 μg/ml) for 72 h and cell viability was estimated by MTT assay. **B. & C.** The cytotoxicity of fraction-174 (Ericifolin purified from AAE by an independent strategy) was tested in both **B.** MCF7 and **C.** MB231 cells using MTT assay. **D.** Ericifolin does not induce apoptosis or autophagy in MDA-MB231 cells. 10,000 cells were plated in 24-well clusters and treated with Ericifolin (50 μg/ml) for 24 h. Cell lysates were made and tested against c-PARP and LC3B for apoptosis and autophagy activity, respectively.

Previously we showed AAE exerted its anti-cancer effect in several prostate cancer cell lines *in vitro* and LNCaP tumors *in vivo*, and identified a compound with anti-proliferative and anti-androgen receptor functions: Ericifolin, [Eugenol 5-O-β-(6′-galloylglucopyranoside)], from AAE [18]. We reported that this is the only cytotoxic compound identified from AAE against prostate cancer cells [18]. Using the same HPLC based purification method [18], we identified 10 fractions with absorption maxima at 254 nm and tested their cytotoxicity on MCF7 cells. The initial cytotoxicity test shown in Figure 6A indicated that Ericifolin (peak No.7 active in LNCaP cells) is not cytotoxic in MCF7 cells. We also tested highly purified Ericifolin in both MCF7 and MB231 cells, no significant cytotoxicity was found (Figure S2A). We next tested preparations of Ericifolin independently purified from a different laboratory (labelled as Fraction 174) with NMR validated structures (Figure S2B & 2C). As shown in Figure 6B & 6C, fraction No.174 showed minimal cytotoxicity in BrCa cells and it was not dose dependent. In addition, Ericifolin was confirmed to not induce apoptosis or apoptosis in BrCa cells (Figure 6D). Lack of activity of Ericifolin on BrCa cells indicated unique compounds in AAE other than Ericifolin that might be involved in autophagy driven cytotoxicity.

### AAE inhibits growth of MB231 tumors in athymic mice

**Figure 7:**
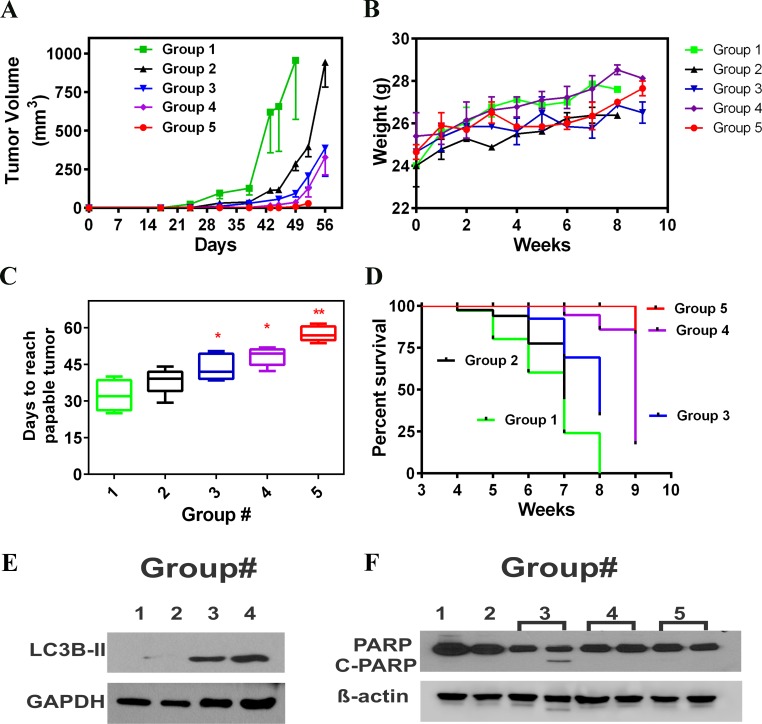
AAE inhibits MDA-MB231 tumors *in vivo* **A.** Tumor growth kinetics of MB231 tumors in mice in all 5 treatment groups: Groups:(1) Vehicle, (2) Daily AAE gavage after tumor cell injections, (3) AAE pre-gavage plus AAE gavage after tumor cell injection, (4) Tumors originated from cells pretreated with AAE but no AAE gavage after *in vivo* injection and (5) Mice injected with pre-exposed cells and then gavaged with AAE daily. (Vertical bars: SD, one sided). **B.** Mean body weight (g) of mice in vehicle and various treatment groups, measured weekly. **C.** Days needed for tumor size to reach 40 mm^3^ for different treatment groups. Description for different groups is shown in Table 1. Non-linear regression fit was generated using Graph Pad Prism for each mice and the days need to reach 40 mm^3^ was calculated using equations accordingly, symbols shown represent mean time needed (vertical bars: SD, *n* = 5-7). **D.** Survival of mice from all five groups. Data selected from week-3 to week-9 (end point of experiment was week 8, except in Group 5 where were euthanized at the end of 9 weeks because, 4 out of 6 mice (76 %) did not have palpable tumor. Time of event is tumor reaching 100 mm^3^ (0.1 cc) in volume. **E.** Increased levels of autophagy were detected in tumor samples of AAE treated mice. Protein was extracted using RIPA lysis buffer. Immunoblotting analysis was done against LC3B antibody. GAPDH was used as a loading control. **F.** Lack of apoptosis (cleaved PARP) in tumor tissues from all five groups. Baring one sample in Group 3, cleaved PARP was not detected in any tumor samples.

We determined the oral bioavailability of AAE *in vivo* by gavage and intraperitoneal injection of AAE and sampling sera from mice. A group of mice were given AAE by IP injection of 300mg/kg for a duration of one to 24h and the total polyphenol contents of sera from treated mice were determined by Folin's method [58]. As shown in Figure S3, the serum polyphenol was highest at 24h after injection. The bioavailability of AAE by oral gavage was determined by dosing the mice for 2 weeks with dose escalation of AAE (100-300mg/kg) yielded similar levels in sera. The highest serum intake of AAE (estimated by total polyphenol) was found in 300mg/kg group, indicating good bioavailability of AAE by oral gavage. Later, 150mg/kg AAE was given to the mice in pre-gavage chemo preventive study group because oral intake of AAE at this concentration was comparable to that with the highest dose (Figure S3) and further, as a measure of non-toxic dose, body weight was steadily maintained for the entire duration of the treatment during tumor formation (Figure 7B).

We investigated the antitumor efficacy of AAE in female athymic Balb/C mice grafted with a 50:50 mix of matrigel and MB231 cells (2 × 10^6^) on their posterior flank. Treatment strategy is shown in Table 1. Mice were randomly assigned to control (group-1) and AAE gavage groups (group-2). The day after tumor cell injection, mice in group-2 were gavaged daily with AAE (150mg/kg). As shown in Figure 7A and Table 1, analysis based on weekly measurement of tumor volumes, and estimating the growth time required by the tumors to grow into a tumor volume of 1,000 mm^3^ showed some tumor growth inhibition in AAE-gavaged animals (~14 %) but not significant. Similarly, there was no significant (*p* = 0.18) increase in mean palpable tumor incidence between two groups. No significant body weight loss was observed in treatment compared to control group (Figure 7B). In an independent experiment, we tested the potential of AAE to prevent tumor incidence or potential delay in the appearance of palpable tumor (group-3). A group of athymic mice received 2 weeks of AAE gavage (150 mg/kg, 7days/week) before we injected MB231 cells and gavage with AAE continued until the termination of treatment (8 weeks). Tumor growth in group-3 was significantly reduced compared to group-2 (Figure 7A, Table1). AAE pre-gavage also delayed the appearance of palpable tumor (volume of ≥ 40 mm^3^) (Figure 7C, Table 1). In group-3, palpable tumor detection occurred at 7^h^ week after cell injection compared with 6^th^ weeks from group-2, and 4.5 weeks in control group (Figure 7C, Table 1). Among the groups where we pretreated the BrCa cells before injection, we observed a significant decrease in tumor burden with AAE gavage (Figure 7A, Table 1). As shown in Figure 7C and Table 1, in group-4, the palpable tumor detection time is 7 weeks (48 days), while addition with AAE gavage further prolonged the mean tumor palpable growth to 58 days. None of the animals in group-5 developed tumors which reached the end-point to euthanize during the duration of the experiment. The overall survival curve showed a clear pattern indicating that AAE gavage, especially in combination with pre-AAE treatment, yield significant survival benefit in the human tumor xenografts (Figure 7C & 7D).

**Table 1:** Summary of growth of MDA-MB231 tumors in female athymic mice with subcutaneous tumor cell injection

Group No. (Gr.)	Treatment	Palpable Tumor Detected (days post injection) (mean ± SD)	Significance Compared to Vehicle^1^	Days to reach ≥ 1,000mm^3^ post injection (mean ± SD)	Significance Compared to Vehicle^1^
1	Vehicle	32.2 ± 6.4	NA	50.7 ± 7.6	NA
2	AAE gavage	38.3 ± 5.4	P=0.18	57.2 ± 3.1	*P* = 0.20
3	Pre-gavage +post-tumor implant gavage	44.2 ± 5.1	*p<0.05	72.1 ± 11.8	*P < 0.03
4	Pre-treated cells without post-gavage	48.2 ± 3.6	**P<0.01	69.3 ± 12.3	*p < 0.02
5	Pre-treated cells with post gavage	57.6 ± 3.1	**P<0.005	92.7 ± 22.6	**P < 0.01

1 Significance were determined by unpaired comparison using Wilcoxon-Mann-Whitney test; Levels of significance, *p ≤ 0.05, ** *p* ≤ 0.01.

To investigate the mechanism of AAE-induced tumor growth inhibition *in vivo*, tumor tissues collected at necropsy were analyzed by western blot for apoptosis and autophagy (LC3B). Consistent with *in vitro* findings, increased LC3B expressions were detected in AAE gavage/AAE pre-gavage/AAE pretreated cells groups compared to vehicle control group (Figure 7E). But no PARP cleavage was detected in either group (Figure 7F).

## DISCUSSION

The above results established that AAE has cytotoxic activities against human BrCa cells *in vitro* and against a triple negative BrCa (TNBC) MB231 model *in vivo*. Moreover, we demonstrated that AAE induced cell death is associated with features of autophagy, but not apoptosis, a common cell death mechanism exerted by most anticancer drugs, including some diet-derived compounds, such as curcumin and capsaicin [37, 38]. Our results also suggest that the induction of autophagy by AAE is related to the inhibition of Akt/mTOR signaling. Most importantly, AAE was shown to be effective in decreasing the growth of MB231 tumors by oral administration, and this effect was even greater when the mice were pretreated with AAE, as a cancer chemo-preventive agent.

The cytotoxicity and colony forming assays showed that well-characterized TNBC model, MB231 tumor cells, exhibit the highest sensitivity to AAE among all BrCa cells tested. MB231 cells are highly metastatic and faster growing than luminal type MCF7 cells. However, the cell cycle studies showed no significant effect of AAE on both cell types, indicating, the DNA replicating difference does not weigh in explaining the difference in sensitivity to AAE. We noticed that MB231 and SKBr3 cells, which are both ER negative (although differing in ERBB2 expression levels), respond to AAE better than the ER positive cells. We think AAE is more effective on fast growing cells without affecting cell cycle distribution. The high efficacy of AAE on TNBC cells suggests potential clinical benefit. TNBC patients generally do well if pathologic complete response is achieved following chemotherapy however, if residual disease exists and progresses to metastasis, the prognosis is worse among the different breast cancer subtypes.

We observed a close association of increased autophagy with increased cytotoxicity of AAE. Furthermore, the inhibition of autophagy decreased AAE cytotoxicity. Autophagy was originally known for its cytoprotective role by its ability to recycle nutrients and maintain cellular homeostasis. However, recent evidence suggests that autophagy can act as a cell death mechanism [39, 40]. Although there is no clear consensus, it is generally recognized that autophagy plays dual-role in cell survival and death. There's no clear definition of the features of autophagic cell death (ACD), and the term itself raised considerable discussion about its accuracy [41, 42]. Recently, the Nomenclature Committee on Cell Death (NCCD) proposed the criteria that the use of the term ‘autophagic cell death’ should only come from a functional perspective and limited to cases where cell death is delayed by inhibition of the autophagic machinery [43]. Although cautious, we base our assertion on the set of criteria defining autophagy as a cell death mechanism [40]: (i) cell death occurs without the involvement of apoptosis; although apoptosis and autophagy are shown to have cross-talks, they are not mutually exclusive and do coincide [44]. We did not observe any sign of apoptosis during AAE-induced cell death in BrCa cells. We saw no sign of apoptosis in AAE induced cell death including, no change in mitochondrial membrane potential, lack of apoptosis associated caspases activation, and the characteristic DNA fragmentation. Autophagy has been mentioned as one type of caspase-independent cell death [45]. Moreover, the fact that lack of ATP depletion up to 24h after AAE treatment (data not shown) makes necrotic cell death unlikely. (ii) There was an increase of autophagic flux; autophagy is a dynamic process that can be modulated both positively and negatively. The increase in the detected marker (LC3B protein or fluorescent-LC3B puncta) may reflect increased autophagosome formation due to increases in autophagy, or to reduced turnover of autophagosomes [24]. In this study, a combination of AAE with lysosomal inhibitors that inhibit downstream lysosomal degradation, increased LC3B level compared to treatment with AAE or inhibitors alone. (iii) Suppression of autophagy via both pharmacological inhibitors and genetic approaches was able to rescue BrCa cells from cell death. Our functional study which inhibited autophagy by silencing ATG7 and ATG5 (key players in autophagosomes formation) rescued BrCa cells from cell death, indicating the pro-cytotoxic role of autophagy in AAE induced cell death. In contrast, chemical autophagy inhibitors such as 3-methyladenine (3-MA) have also been shown to rescue cell death in other systems, but not in our studies.

Interestingly, we found combination of 3-MA and AAE resulted in an increased cell death in BrCa cells. This might be due to the dual role of 3-MA on autophagy, for instance, 3-MA is shown to promote autophagy when cells are treated in serum containing medium [46], or due to the low specificity of autophagy inhibitors: 3-MA was found to have different temporal roles on class I and III PI3K, and the autophagy inhibitory role was based on its inhibition of class III PI3K [46].

Many studies have shown that the AKT/mTOR pathway is critical in the regulation autophagy. AAE greatly inhibited AKT phosphorylation as well as mTOR phosphorylation. Noticeably, AAE not only down-regulated the phosphorylation of AKT at Thr_308_, which is involved with mTORC1 activation, but also on the Ser_473_ site which is shown to be phosphorylated by mTORC2 [47]. There has been evidence showing that selective mTORC1 inhibition can elicit increased AKT S_473_ phosphorylation and attenuates the signaling effect on tumor cell proliferation [48, 49]. In this case, AAE significantly decreased both sites of AKT which are responsible for the activity of AKT [32] but also overcome the potential re-activation of AKT at Ser_473_ by mTORC1 inhibition [50]. This inhibition effect was accompanied by the decrease in phosphorylation of S6K and increase in p-4EBP1, the downstream substrates of mTORC1 and key regulators in cell growth, proliferation and survival [6]. In addition, we showed that down regulation of mTOR pathway by AAE may explain the autophagy induction. The ULK1(mammalian homologue of ATG1), ATG13, FIP200 complex was shown to be the node for integrating autophagy signals into autophagosome biogenesis [36] and the direct phosphorylation of mTOR and AMPK on ULK1 was shown to regulate autophagy [35]. Consistently, we found a decrease in phosphorylation of ULK1 Ser_757_, which indicated ULK1 activation in AAE treated MCF7 cells, however, no such change was observed in MB231 cells. This might be because of the activation of ULK1 results from changes in different phosphorylation sites. Furthermore Ser_757_, Ser_317_ and Ser_777_ of ULK1 are also shown to be the direct phosphorylation sites for AMPK whose increase indicates the activation of autophagy under glucose deprivation. Although no change in phosphorylation of ULK1 was found in MB231 cells, we did observe the increase of ATG13, which is another player in the complex bridging mTOR and autophagy. We think both changes from ULK1 and ATG13 served as evidence implying the role of Akt/mTOR cascades in AAE mediated autophagy induction in BrCa cells. The change of cellular energy status sensed by AMPK is a well-known major upstream candidate for the mTOR mediated autophagy induction, however, we did not detect the activation of AMPK or ATP depletion in AAE treated BrCa cells (data not shown), suggesting intracellular metabolic stress is not the cause for autophagy induction. Thus, our study suggests that the down regulation of Akt by AAE treatment caused mTOR inhibition which led to autophagy induction.

We are aware that many of the activities on tumor cell cytotoxicity of AAE may or may not be only due to one single compound present abundantly in AAE. AAE is a rich source of many glycoside-containing complex polyphenols, including Ericifolin, gallyol pedunculagin, penta-O-galloyl-β-glucose, simple polyphenol such as gallic acid, elegiac acid, Eugenol, and many others, some of which are not well characterized [51-53]. Our preliminary attempts to reproduce autophagic cell death in BrCa cells using compounds either commercially available, or prepared in our laboratory were not successful. Many of the known compounds mentioned above induce apoptosis of BrCa cells, but no evidence of autophagy. It is also possible that a combination of one or more of already known compounds present in AAE might induce the cytotoxic effect by activating autophagy. Identification of active compound (s) that is cytotoxic to BrCa cells by induction of autophagy is ongoing in the authors' laboratory (data not shown).

The antitumor and chemo-preventive activity of AAE by oral gavage in MB231 model suggests a potential clinical application of AAE. Oral gavage of AAE alone showed significant antitumor activity, which was enhanced when host-mice or tumor cells were pretreated with AAE before injection, resulting in a more significant reduction in tumor formation and growth. Importantly, we were unable to detect any overt toxicity of AAE throughout treatment at 150 mg/kg. In the group of pre-treatment (chemoprevention) plus continuous AAE gavage, most mice (4/6) showed smaller than palpable tumors (<40mm^3^) throughout the duration of the experiment. The mechanism of antitumor activity correlated with *in vitro* observations and was attributed to autophagy, without any detectable evidence of apoptosis.

It is to be noted that the results presented in this report suggest potential synergy of AAE with rapamycin (Figure 4F). Rapamycin has been shown to be both chemo-preventive and anti-senescence compound when taken daily at a low, non-immune compromising dose [54, 55]. The main mechanism of rapamycin even at low concentration is inhibition of mTOR. Therefore, a combination dose of AAE and rapamycin daily may contribute to longer delay in tumor recurrence of BrCa patients and might suggest novel therapeutic avenue for prevention of cancer and aging. However, considering the poorly defined composition of AAE used in our experiments, present a significant challenge to any definitive suggestion for their human use at present.

Allspice is widely used in the Central American cuisines as a seasoning spice for meat cooking or dessert making. Although used as folk medicine in the Caribbean and South America for a long time, few studies have looked into the anticancer potential of Allspice [56]. No epidemiological evidence for Allspice consumption with cancer risk reduction exists. Previous work on prostate cancer reported by us perhaps, the only such study, showed berries of *Pimenta dioica* show anti-cancer property [18]. It should be noted however, the mechanism of anticancer activity reported in the previous study pertains to unique phenotype of prostate cancer, and the cytotoxicity was clearly due to induction of apoptosis and inhibition of androgen receptor transcription. Unique yet different mechanism of anticancer activities of AAE on two major human diseases, prostate and breast cancers promises AAE to possess great potential as a dietary supplement for cancer chemoprevention.

## MATERIALS AND METHODS

### Preparation of AAE

A water extract of Certified-organic *Pimenta Dioica* berries (Oregon Spice Company, Portland, OR) were prepared as reported [18] and used in all studies presented here. Briefly, a water extract of berries or powdered Allspice prepared by boiling for 10 min at 10 % w/w, filtering the extract through Whatman #1 paper, concentrating by lyophilization and re-dissolving the freeze-dried sample at suitable concentration for studies reported here. AAE thus extracted has been consistently found to contain polyphenols at 38 %, carbohydrates (15 %), amino acids (1 %) etc., as detailed elsewhere [18].

### Cell lines and reagents

All human BrCa cell lines (MCF7, MDA-MB231, SKBr3, BT474, and T47D) and non-malignant breast epithelial cell lines MCF-10A and MCF-12A were obtained from the American Type Culture Collection (Manassas, VA). Cells were cultured in RPMI 1640 (Corning Inc., New York) and supplemented with 10 % fetal bovine serum (FBS) and 20 μg/ml gentamicin at 37°C and 5 % CO_2_. MCF10A cells were cultured in a mammary epithelial basal medium (MEBM, Invitrogen Inc., Carlsbad, CA) supplemented with growth factor cocktail SingleQuots™ Kit (LONZA Group, Switzerland). All cell lines used in this study were authenticated for their origin and genetic composition (Genetica DNA Laboratories Inc. Cincinnati, OH). All cell cultures were routinely tested against potential mycoplasma contamination and only mycoplasma-free cultures were used in all experiments.

### Cell proliferation and clonogenic assay

Cell viability was assessed by the methyl thiozolyl tetrazolium bromide (MTT) reduction assay and by cell counting with trypan-blue exclusion. MCF10-A cells were cultured in MEBM supplemented with 0.1 % BSA for 24h of starvation, then exposed to various concentrations of AAE for 72 h in both basal and growth-factor containing medium for cell viability test. For clonogenic survival assays, BrCa cells were plated at low density (500-1,000 cells/35mm dish) following treatment with several concentrations of AAE for 24h, washed, and replace with fresh complete medium and then allowed to form individual colonies. Colonies were stained and fixed in 1 % crystal violet after 10-12 days of culture and were manually counted with colony criteria as described before [18].

### Transfection

siRNA transfection: siRNA ATG7, siRNA ATG5, and siRNA control were purchased from Thermo Fisher Scientific Inc. MB231 and MCF7 and MDA-MB231 cells were seeded (1*10^5^ cells per well) in 6-well plate RMPI 1640 supplemented with 10 % FBS without antibiotic and left to attach for 24h, and were transfected with 25nM siRNAs (Dharmafect On-target Plus) using Dharmafect 2 transfection reagents (Thermo-Fisher Scientific, Waltham, Massachusetts, MA, USA). Transfected cells were harvested and used for subsequent experiments 48h later.

### Plasmid (mCherry-GFP-LC3B) DNA transfection

The mCheery-GFP-LC3B plasmid (AddGene Inc., Cambridge, MA) was transfected into MCF7 cells according to the manufacturer's protocol (Lipofectamin 2000 for MCF7, Life Technologies). About 4 × 10^4^ cells/250μl were plated into 8-chambered glass slide (LabTek Inc.) and incubated for 24h before 0.8μg of plasmid diluted with Lipofectamin 2000 reagent (1:1 ratio) was transfected into the cells. Transfection efficiency was evaluated under microscopy for GFP signal after 12h, and transfected cells were used for subsequent experiments 48hs later.

### Immunoblotting

Lysates from AAE-treated and AAE-untreated cultures were prepared with cell lysis buffer, fractionated and analyzed by immunoblotting [18]. Polyclonal anti-PARP, anti-LC3B, anti-AKT, anti-mTOR, anti-phospho-mTOR (Ser2448), anti-S6 ribosomal protein, anti-phospho-S6 ribosomal protein (T389), anti-4EBP1, anti-phospho-4EBP1 (Thr37/46), as well as anti-ULK1 and anti-phospho-ULK1 (S757) antibodies were purchased from Cell Signaling Technology. Polyclonal anti-phospho-AKT (Ser473, T308) antibody was purchased from Epitomics (now Abcam Inc., Cambridge, MA). GAPDH antibody was purchased from ProteinTech Group Inc. (Chicago IL). Equal loading of cell lysates in western blots were ensured by loading equal amounts of total cell proteins or cell equivalents by performing either cell counts prior to solubilization with SDS-PAGE gel sample buffer or by comparing the band densities of specific proteins with that of β-actin. In blots that have molecular mass close to that of β-actin, identical gel was run with same cell lysates and immunoblotted for β-actin.

### Analysis of cell morphology

MDA-MB-231 cells were seeded in four-well Lab-Tek Chamber slides (Thermo Fisher Scientific) at 10^6^ cells/ml for 24h. Cultures were then incubated with AAE (25-75μg/ml) or 100 nM rapamycin (Sigma-Aldrich Inc., St Louis, MO) for 24h. Cells were washed with PBS and mounted with Vectashield mounting medium (Vector Laboratories., Burlingame, CA) and imaged under a phase-contrast microscope.

### Fluorescence confocal microscopy

BrCa cells were treated with AAE for 24h and the detection of LC3B expression was conducted as described [57]. The pattern of endogenous and induced expression of Green Fluorescence-LC3 was observed using AxioVision microscopy software (Zeiss Inc. NY).

For autolysosome detection by mCherry-GFP-LC3 transfection: 48h after plasmid transfection, cells were treated with AAE for 12h and changed with fresh medium. Then cells were washed and mounted with Vectashield mounting medium with DAPI and visualized suing Carl Zeiss Scanning Confocal microscopy (Zeiss Inc. NY).

### Serum polyphenol estimation for AAE bioavailability

24 C57-B6 mice (Harlan Labs, Indianapolis, IN) were randomly assigned to 2 groups. First group of mice received IP injection of 300mg/kg AAE for several time points (1, 3, 6, 8, and 24h). Second set with two mice each received gavages of AAE (100, 150,200, and 300 mg/kg) daily for 2 weeks. Blood was drawn at the end of treatment, centrifuged at 15,000g for 15min. and 10μl of serum was taken and mixed with Methanol at 1:2 ratio. This solution was mixed for 1 min in a vortex mixer, centrifuged at 1,100X g for 15min and the supernatant was used for polyphenol estimation [58]. Briefly, gallic acid (10mg/ml) (Sigma-Aldrich Inc.) was used as a standard to estimate the total polyphenol in serum using 1:1 Folin-Denis' reagent (Sigma-Aldrich Inc.) and 7.5 % sodium carbonate. Samples were incubated at room temperature for 30min and 0.1 ml was transferred into wells of 96-well clusters and read at 765nm (Benchmark Plus Plate Reader, Bio-Rad Laboratories, Inc. Richmond, CA). Polyphenol concentrations were calculated based on a standard curve generated using Gallic acid as a standard.

### Antitumor activity *in vivo*

All experiments were carried out using an institutional animal care and use committee (IACUC) approved protocol. Number of animals per treatment group was determined by power analysis as described before (18). Six to eight week-old athymic female mice (Harlan Labs, Indianapolis, IN, USA) were injected with a suspension of MB-231 cells (2×10^6^ cells/200μl/mouse) mixed with solubilized basement membrane extract (pathogen-clear BME, Trevigen, Gaithersburg, MD) at 1:1 ratio, at a subcutaneous site on the upper left flank. Mice were gavaged daily with a solution of AAE in water (150mg/kg). For testing the chemoprevention potential of AAE, mice were gavaged AAE daily for 15 days before injecting tumor cells. We pretreated the MB231 cells with AAE *in vitro* for 48h and injected approximately same number of viable cells (2 × 10^6^) into mice. The mice were then randomized to a control (group-4) or continuous AAE gavage (150 mg/kg) group [group-5]. Tumor growth was monitored over time by measuring tumor volume twice a week with a hand held caliper and volume estimated as described before (18). Tumors were considered palpable if a firm nodule was palpable but was <3mm in diameter. Mice were weighed weekly to identify any potential systemic toxicity of AAE or changes due to tumor growth. Systemic toxicity was also monitored by histologic appearance of any abnormality in liver, lung, heart, and kidney harvested at the time of necropsy.

### Statistical analysis

All numerical data presented are analyzed for statistical significance with probability of accepting null hypothesis with ≤0.05 (*p* ≤ 0.05) using Student's t-test or one way ANOVA. Data obtained from tumor growth *in vivo* were analyzed and tested the inter group significance by Wilcoxon-Mann-Whitney test. Qualitative data presented in western blots and micrographs are representative of at least two independent determinations, one representative set of data are presented.

## SUPPLEMENTARY MATERIAL FIGURES


