# Supplementary material for: Polyphenol-rich extract of Pimenta dioica berries (Allspice) kills breast cancer cells by autophagy and delays growth of triple negative breast cancer in athymic mice
Source: Oncotarget. 2015 Apr 14;6(18):16379–95. doi: 10.18632/oncotarget.3834 (PMC4599276; doi:10.18632/oncotarget.3834)
Supplement: Supplementary file 1 [file oncotarget-06-16379-s001.pdf]

Polyphenol-rich extract of *Pimenta dioica* berries (Allspice) kills breast cancer cells by autophagy and delays growth of triple negative breast cancer in athymic mice

Supplementary Material

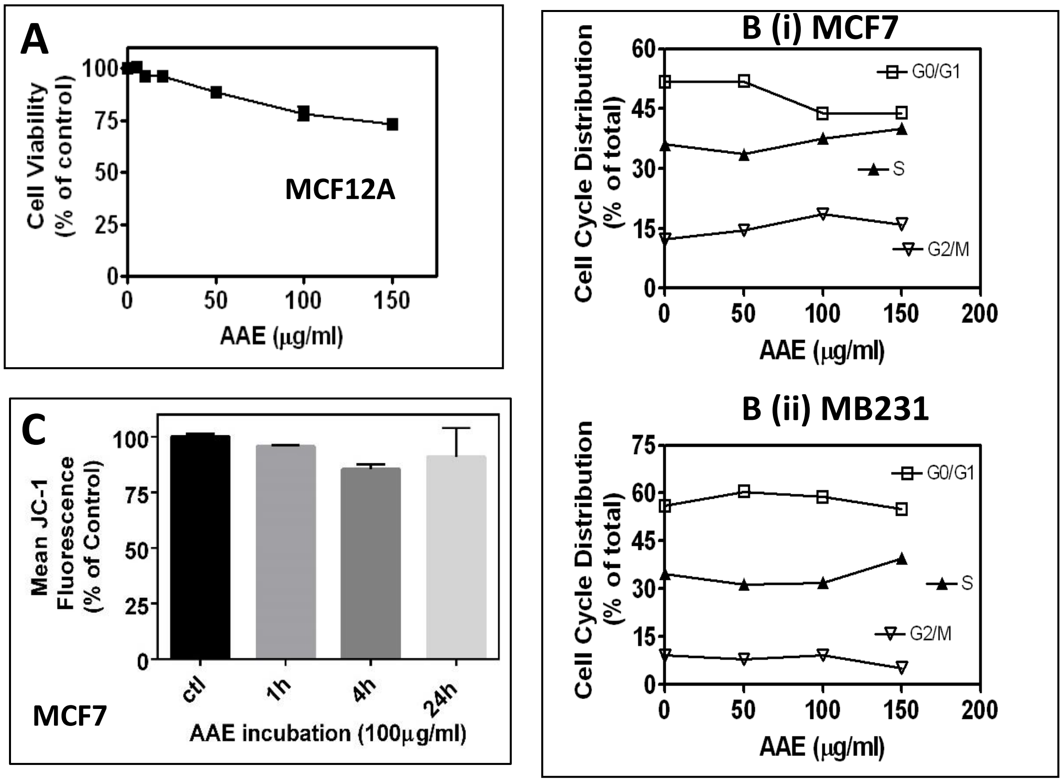

Figure S1: **(A)** AAE has limited cytotoxicity in untransformed-human mammary-epithelial MCF-12A cells. Viability decreased by 25% after 72h exposure at the highest concentration (150 μg/ml); **(B)** AAE treatment does not affect cell cycle distribution in BrCa cells. Propidium iodide stained nuclei from ~3 x 10^5 BrCa cells exposed to several concentrations of AAE for 24 h were analyzed by flow cytometry for DNA content to determine cell cycle phase-fractions as described before (18).Percent of cells distributed into each of the three phases are presented in B(i) for MCF-7 and B(ii) for MB231 cells after analyzing the amount of PI bound to the DNA using Modfit program (18). **(C)** AAE treatment does not change the mitochondrial depolarization as shown by JC-1 green fluorescence intensity. About 1 x 10^5 MCF7 cells were plated and treated with AAE up to 24h. Cells were loaded with JC-1 (MitoCapture Reagent, Abcam) for 30min in 37°C incubator for 30min. Cells were analyzed by flow cytometry using FITC channel for green fluorescence whose increase indicates apoptosis. Mean value of green fluorescence intensity are shown.

A

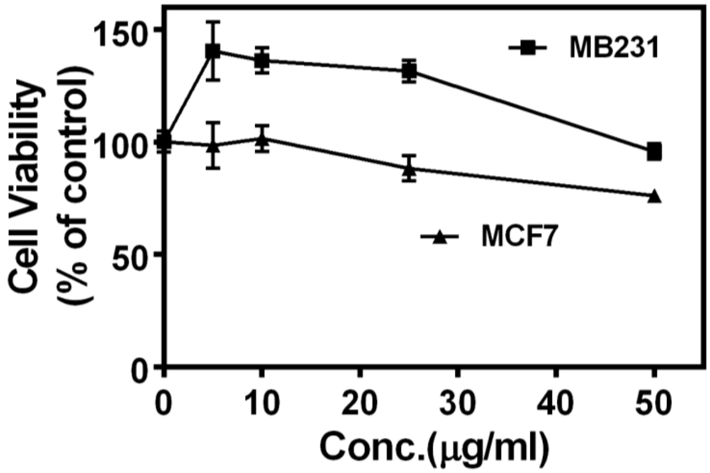
MB231
MCF7
Cell Viability
(% of control)
150
100
50
0
0
10
20
30
40
50
Conc.(μg/ml)

B

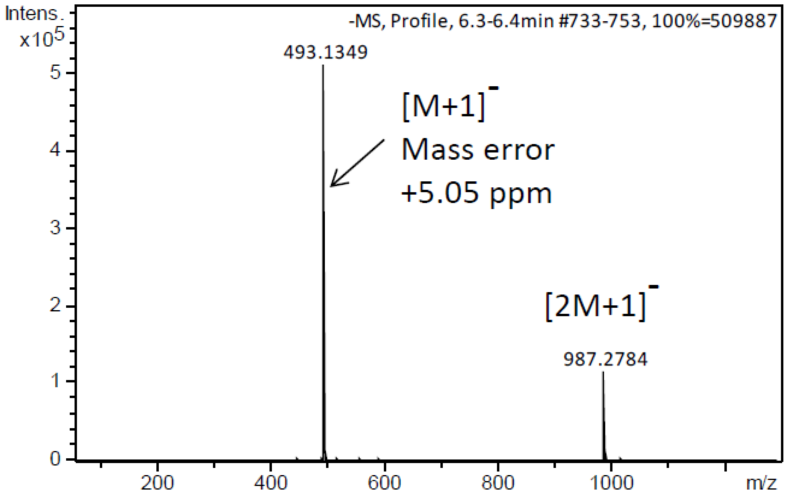
Intens.
x10^5
-MS, Profile, 6.3-6.4min #733-753, 100%=509887
493.1349
[M+1]-
Mass error
+5.05 ppm
[2M+1]-
987.2784
5
4
3
2
1
0
200
400
600
800
1000
m/z

C

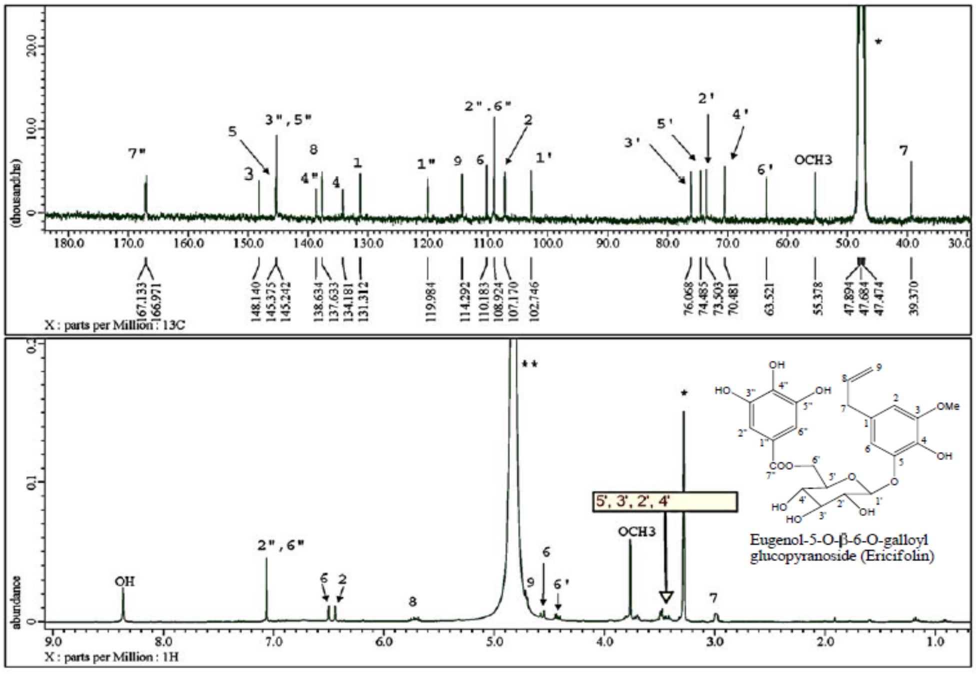
7"
5
3",5"
3
8
4"
4
1
1"
9
6
2".6"
2
1'
3'
5'
2'
4'
6'
OCH3
7
*
167.133
166.971
148.140
145.375
145.242
138.634
137.633
134.181
131.312
119.984
114.292
110.183
108.924
107.170
102.746
76.068
74.485
73.503
70.481
63.521
55.378
47.894
47.684
47.474
39.370
X : parts per Million : 13C
(thousandths)
**
OH
2",6"
6
2
8
9
6'
OCH3
5', 3', 2', 4'
abundance
X : parts per Million : 1H
Eugenol-5-O-β-6-O-galloyl
glucopyranoside (Ericifolin)

Figure S2: Ericifolin is not cytotoxic to BrCa cells and the Revalidated structure of Ericifolin. (A) The cytotoxicy of Ericifolin (Purified from AAE in our lab) was tested in both MCF7 and MB231 cells using MTT assays. (B) High resolution mass spectrum of Ericifolin in negative ionization mode (C) ^{13}C (top) and ^{1}H NMR(bottom) spectra of Ericifolin recorded at 100 and 400 MHz respectively in methanol-d_4, * denotes residual MeOD and **residual water signals from MeOD. Assignments of all carbon and proton resonances were made using 2D HMQC and HMBC spectra.

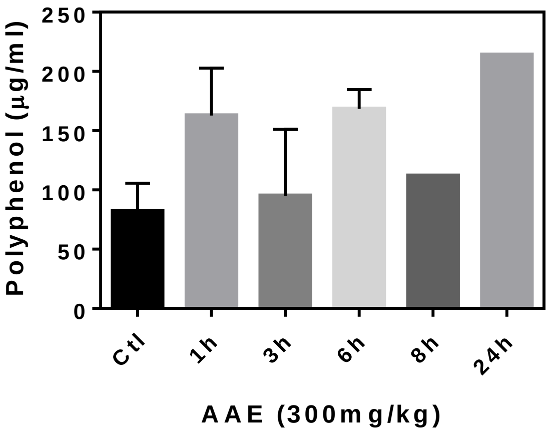

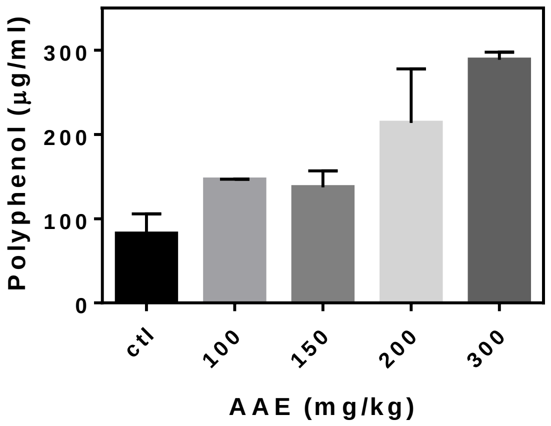

Figure S3: The bioavailability of AAE detected by polyphenol estimation from mice serum. Upper: Levels of total polyphenol in sera of mice given IP injection of AAE (300mg/kg) for 1-24h. Bottom: Serum polyphenol levels in mice that received oral gavage of AAE (100-300mg/kg) for 2 weeks. Gallic Acid (10mg/ml) was used as a standard for polyphenol estimation. Mean values of estimated polyphenol in the serum. Serum polyphenol levels increased due to duration of treatment or the amount used in each dosing.
